# An IMU-to-Body Alignment Method Applied to Human Gait Analysis

**DOI:** 10.3390/s16122090

**Published:** 2016-12-10

**Authors:** Laura Susana Vargas-Valencia, Arlindo Elias, Eduardo Rocon, Teodiano Bastos-Filho, Anselmo Frizera

**Affiliations:** 1 Postgraduate Program in Electrical Engineering, Federal University of Espirito Santo (UFES), 29075-910 Vitória-ES, Brazil; laura.valencia@aluno.ufes.br (L.S.V.-V.); teodiano.bastos@ufes.br (T.B.-F.); frizera@ieee.org (A.F.); 2 Postgraduate Program in Biotechnology, Federal University of Espirito Santo (UFES), 29040-090 Vitória-ES, Brazil; arlindo.elias@aluno.ufes.br; 3 Neural and Cognitive Engineering Group, Centro de Automática y Robótica, CSIC-UPM, 28500 Madrid, Spain

**Keywords:** inertial sensor, joint angular kinematics, human motion analysis, anatomical calibration, technical frames

## Abstract

This paper presents a novel calibration procedure as a simple, yet powerful, method to place and align inertial sensors with body segments. The calibration can be easily replicated without the need of any additional tools. The proposed method is validated in three different applications: a computer mathematical simulation; a simplified joint composed of two semi-spheres interconnected by a universal goniometer; and a real gait test with five able-bodied subjects. Simulation results demonstrate that, after the calibration method is applied, the joint angles are correctly measured independently of previous sensor placement on the joint, thus validating the proposed procedure. In the cases of a simplified joint and a real gait test with human volunteers, the method also performs correctly, although secondary plane errors appear when compared with the simulation results. We believe that such errors are caused by limitations of the current inertial measurement unit (IMU) technology and fusion algorithms. In conclusion, the presented calibration procedure is an interesting option to solve the alignment problem when using IMUs for gait analysis.

## 1. Introduction

Functional mobility usually refers to the skill of ambulating safely in a free living environment by walking, running, climbing and even when handling assistive devices, such as walkers, crutches and canes [1,2]. About 15% of the world’s population live with some disability condition, of which 2%–4% suffer significant functional problems [3]. In this scenario, one of the major goals of neuromuscular rehabilitation is to regain gait function in order to promote more independent lives [4]. The assessment of functional activities, such as walking, can help clinicians to determine patients’ autonomy level and the optimal care they should receive [5].

Therefore, it is essential to understand and characterize systematically motion disturbances to improve the diagnosis, enhance treatments and measure patients’ evolution. The estimation of joint angular displacements is a fundamental part of human motion analysis and involves the detection of joint position and spatial orientation [6]. The relevance of these parameters is observed in many clinical scenarios such as gait training and rehabilitation in patients with stroke, Parkinson’s disease and cerebral palsy [7,8,9].

In contrast to camera-based laboratory systems for measuring joint angles, wearable sensors present advantages of lower cost, higher flexibility, portability and adaptability [6,10]. This is the case of inertial measurement units, commonly referred to as IMUs or inertial sensors. These sensors are a multi-axial combination of accelerometers, gyroscopes and eventually magnetometers, which can be attached to different body segments to estimate joint kinematics. Considering their usability in internal and external environments and fast donning and doffing, these sensors represent a promising technology that may become an alternative to high-cost optical systems [11,12,13,14,15]. 

This topic has evolved into a wide and solid field of research, but clinical applications involving the use of IMUs are still largely unexplored in the literature. Perhaps this is due to the lack of standards for placing sensors on body segments and defining joint coordinate systems (JCS), which limits the correct calculation of joint kinematics. Also, some studies have questioned the accuracy of these systems [16,17,18,19,20]. Researchers have stated that the calibration stages of the individual sensors (i.e., accelerometer, gyroscope, and magnetometer), biases, sensibilities and different noise types, in addition to sensor fusion algorithm issues, influence the accuracy of the orientation estimation.

Regarding these situations, a fundamental problem of the IMU-based gait analysis is to how define an appropriate measurement protocol and provide a sensor-to-body calibration procedure [21]. Because IMUs’ local frames are not aligned with anatomically defined frames, different approaches in the literature have presented different methods to determine the sensor frame’s orientation with respect to the body segment frame [13,14,15,22]. However, those approaches suffer from some limitations. One main problem with algorithms based only on data from accelerometers and gyroscopes [11,13,23,24] is the difficulty to define a common reference frame and, consequently, measure 3D angles. To accurately measure 3D angles, a second global reference axis is necessary along with the gravity vector. This second reference axis is commonly the magnetic field vector, measured by sensor units that include magnetometers. Since heading drift remains a problem within systems that involve only accelerometers and gyroscopes, the anatomical calibration techniques that use such systems rely on predefined user movements to define the axis of joint motion [11], or use supplementary devices such as cameras [24], anatomical landmark pointers [12] or exoskeleton harnesses [13]. The need for these additional tools also increases the experiment duration and requires experienced personnel, which may be impractical in daily clinical routine.

Other works are based on performing complex movements while keeping some specific postures [13,14,22], or maintaining the same orientation or joint angle between two postures [15,24], which may not be simple tasks if to be performed by subjects with motor disabilities. Even for subjects without disability, performing these tasks requires the assistance of examiners. Hence, these mentioned methods may be more prone to calibration errors.

The objective of this work is to present a novel calibration procedure as a method to align IMU sensors to body segments, which compared to the aforementioned methods, is based on fast and simple sensor placement procedures, with no need for movements performed by the user nor any additional tools. Initially, we propose a validation protocol of the procedure using a simplified rigid-body joint that comprises two semi-spheres. A universal goniometer is used as the gold standard measure in order to ensure controlled angular movements. Additionally, we present an application of the method on five able-bodied subjects performing a gait test. The kinematic data of the lower limb joints is presented descriptively.

This paper is organized as follows: Section 2 describes the proposed IMU-to-body alignment method that includes the calibration algorithm, definition of technical-anatomical frames and calculation of joint angles. Then, in Section 3 we present the motion acquisition system and the validation protocol using the simplified joint, along with an evaluation procedure to quantify the accuracy and repeatability of the technique. Following, a sensor placement protocol and an estimation of kinematic data on subjects without functional disability are introduced in Section 4. Finally, we provide the results and discussion of the experiments that validate the proposed method (Section 5), followed by the conclusions (Section 6).

## 2. IMU-to-Body Alignment Method

To estimate the lower limb joint angles, it is necessary to measure the orientation of two adjacent body segments forming the joint. We propose a method to estimates hip, knee and ankle joint angles of the lower limb. To simplify the mathematical explanation, here we only present the data for the right leg, although the same concept may be obviously applied to both legs. In this method, four IMUs sensors are used: one is placed on the pelvis (body segment named PV), one on the right thigh (TH), one on the right shank (SH), and another on the right foot (FT). Each body segment also has an associated coordinate system (BF), which is called, in this work, a *technical-anatomical frame*. Note that the technical-anatomical frame is an estimate and it is also different from the anatomical bone-embedded frame as defined by the International Society of Biomechanics (ISB) recommendations [25,26]. The reason is that the axes of body segments’ Cartesian coordinate systems, within ISB recommendations, are defined based on bony landmarks that are palpable or identifiable from X-rays.

### 2.1. Calibration Algorithm and Definition of Technical-Anatomical Frames

During five seconds of static acquisition (initial upright posture), the orientation data is used to define the sensor-to-body alignment. The first stage consists of correcting the sensor frame placed on the pelvis (called IMU-F-PV coordinate system). This correction procedure aims to align the IMU-F-PV with the gravity. Let qIMU−F−PVOGF be the quaternion of the IMU placed on the pelvis, in the initial posture computed by averaging the orientation data (as in [27]) acquired over 5 s interval. Since the orientation data is obtained in quaternion format, the operations to align the sensor quaternion qIMU−F−PVOGF with the gravity are performed as follows:

(1) Obtain *x*-axis ($x_{IMU-F-PV}$) of the coordinate system referred to the IMU orientation measured by the quaternion qIMU−F−PVOGF associated with the initial posture, and using Equation (1) to convert from unit quaternions to direction cosine matrix, $x_{IMU-F-PV}$ defined as shown in Equation (2):
  (1) $$M(q)=[\begin{array}{ccc} q_{0}^{2}+q_{1}^{2}-q_{2}^{2}-q_{3}^{2} & 2(q_{1}q_{2}-q_{0}q_{3}) & 2(q_{1}q_{3}+q_{0}q_{2})\\ 2(q_{1}q_{2}+q_{0}q_{3}) & q_{0}^{2}-q_{1}^{2}+q_{2}^{2}-q_{3}^{2} & 2(q_{2}q_{3}-q_{0}q_{1})\\ 2(q_{1}q_{3}-q_{0}q_{2}) & 2(q_{2}q_{3}+q_{0}q_{1}) & q_{0}^{2}-q_{1}^{2}-q_{2}^{2}+q_{3}^{2} \end{array}],$$
  (2) xIMU−F−PV=M(qIMU−F−PVOGF)i,
  where i is the unit vector in direction of the *x* axis.
(2) Define the angle θ between $x_{IMU-F-PV}$ and the gravity vector ***ZG***. The angle θ is calculated using Equation (3):
  (3) $$\theta =\mathrm{acos}\left(2\left(q_{1}q_{3}+q_{0}q_{2}\right)\right),$$
  where $q_{0},\;q_{1},\;q_{2}\;\mathrm{and}\;q_{3}$ are the components of the quaternion qIMU−F−PVOGF.
(3) Define the vector $n_{1}$ orthonormal to the mentioned vectors ($x_{IMU-F-PV}$ and ***ZG***). Around this vector a rotation θ is made according to Euler’s rotation theorem. The orthonormal and unit vector $n_{1}$ is defined as shown in Equation (4). The correction quaternion $q_{c}\left(\theta ,n_{1}\right)$ is calculated using Equation (5):
  (4) $$n_{1}={\left[\begin{array}{ccc} 2\left(q_{1}q_{2}+q_{0}q_{3}\right) & q_{2}^{2}+q_{3}^{2}-q_{0}^{2}-q_{1}^{2} & 0 \end{array}\right]}^{T},n_{1}=\frac{n_{1}}{\left\|n_{1}\right\|},$$
  (5) $$q\left(\theta ,\;n\right)=\left(\cos\left(\frac{\theta }{2}\right),n\;\sin\left(\frac{\theta }{2}\right)\right).$$

The technical-anatomical frame of the pelvis (BF-PV) calculated with respect to the global frame (GF), during the initial posture, is defined as shown in Equation (6):

(6) qBF−PVOGF=qc⊗qIMU−F−PVOGF.

Other initial technical-anatomical frame (BF) using quaternions are defined during the calibration procedure as shown in Table 1.

**Figure 1:**
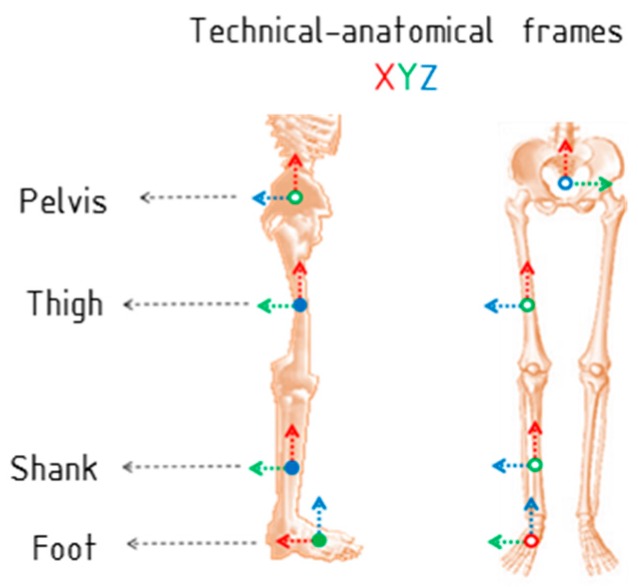
Technical-anatomical frames (BF) of the pelvis, thigh, shank and foot. Axes X, Y and Z in color red, green and blue, respectively.

Once the initial technical-anatomical quaternions are defined, the sensor-to-body orientation qIMU−F−BBF is determined for each sensor using Equation (7):

(7) qIMU−F−BBF−B=qBF−BO*GF⊗qIMU−F−BGF,

where B denotes the body segment, namely PV, TH, SH and FT, and * denotes the complex conjugate of the quaternion. Once we have the relative orientation of the sensor to the body segment, the orientation of each segment at any instant of time can be determined as qBF−PVGF, qBF−THGF, qBF−SHGF and qBF−FTGF, for the pelvis, thigh, shank and foot, respectively. Then, the hip, knee and ankle joint rotations are defined by relating the orientation of the distal body segment with respect to the proximal body segment. The technical-anatomical frames are presented in Figure 1 for each body segment. During the initial posture the joint angles are assumed to be zero, since the corresponding body segments are aligned. The proposed algorithm is also conceived in such a way that the IMUs can be placed in any arbitrary position on the body segments. This means that the user does not have to be concerned about placing the IMUs in an exact position. The algorithm to extract the joint angles is presented in detail in the next section.

### 2.2. Joint Angles Calculation

**Figure 2:**
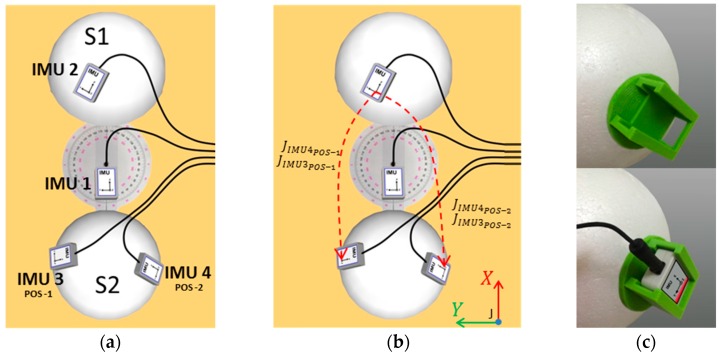
Scheme of a simplified joint comprising two semi-spheres. (**a**) Adjacent segments S1 and S2, and a universal goniometer (a controlled joint J); (**b**) representations of the joint J and (**c**) rigid plastic piece to fit the sensors in a fixed position on the semi-sphere.

The last general reporting standard for joint kinematics based on Joint Coordinate System (JCS) was presented by the International Society of Biomechanics (ISB) [26]. The concept of JCS was first presented by Grood and Suntay [25] only for the knee joint, but this approach has been adopted to define the kinematics of other human joints. This concept uses the description of Cartesian coordinate systems and vector algebra to define the knee joint. In this work, we present the equivalent algebra using quaternions to define hip, knee and ankle joints. Following the method proposed by Grood and Suntay, we compute the body fixed axes and the reference axes of the JCS in Table 2 according to the frames shown in Figure 2. Table 3 summarizes the sign convention used on defining the clinical rotations, where flexion, abduction and internal rotation movements are positives.

Now, let qBF−PVGF, qBF−THGF, qBF−SHGF and qBF−FTGF be the orientation quaternions that represent the frames fixed in each bone. Each body fixed, floating and reference axes, in Table 2, are computed as function of quaternions. Let $e_{2-H}$, $e_{2-K}$ and $e_{2-A}$ be the floating axis of the hip, knee and ankle joint, respectively. The corresponding equations are shown in Equation (8), where |·| indicates that the vector must be normalized, and i, j and k denote the unit vectors in direction of the *x*, *y* and *z* axes, respectively. Then, the equivalent equations in quaternions for calculating the joint rotations are presented in Table 4.

(8) e2−H=(M(qBF−THGF)i)×(−M(qBF−PVGF)j)|·|,e2−K=(M(qBF−SHGF)i)×(−M(qBF−THGF)k)|·|,e2−A=(M(qBF−FTGF)k)×(M(qBF−SHGF)k)|·|,

## 3. Validation Protocol of Calibration Procedure Using a Simplified Rigid-Body Joint

### 3.1. Motion Acquisition System

The motion capture system Tech MCS (Technaid, Madrid, Spain) was used in the experimental procedure. The device was connected via Bluetooth to a laptop. In this study four Tech-IMU V 3.0 sensors were used to obtain orientation data in real-time. Each IMU integrates three different types of three-axial sensors: accelerometers, gyroscopes and magnetometers. Data were acquired using Tech MCS Studio software, which provides orientation (based on Kalman filtering) in quaternion format at 50 Hz. MATLAB software (The MathWorks Inc., Natick, MA, USA) was used to analyze and process the orientation data.

### 3.2. Experimental Procedure

A set-up (Figure 2a) was built with two semi-spheres and the joint angles were measured by an expert physiotherapist using a universal goniometer (360°, 20 cm clear plastic goniometer). Each semi-sphere is used to represent body segments, and the universal goniometer is used to represent an articulation with one degree of freedom.

Using the universal goniometer as reference for measurements, angular movements can be performed in a controlled approach. The rigid semi-spheres are named as S1 and S2 (upper and bottom respectively, see Figure 2a). The joint represented by the goniometer is denoted as J. Rotations from 0° to ±80° with steps of ±20° about *z*-axis of J were performed. These angles correspond to rotations of S2 with respect to S1, which was kept static. The rotation range reaches (or even exceeds) a complete range of motion in lower limbs during walking.

One sensor (IMU 1) was placed on the goniometer, and the three others (IMUs 2, 3 and 4) were placed on two semi-spheres. The IMU 1 is used as the reference, in the same way as the sensor placed on the pelvis for the experiments with human subjects. This sensor also remained static. For each semi-sphere, technical frames were defined as described in Section 2.1 and the equations applied are analogous to those for calculating knee joint angles as described in Section 2.2. Observe that the segment S2 has two sensors (IMUs 3 and 4), that means the technical frame of S2 can be determined using both sensors. Also, these sensors were fixed to the semi-sphere using a rigid plastic pieces made by a 3D printer (Figure 2c), which were glued fitting on the semi-sphere surface. These pieces ensure that the sensors have the same posture when they are exchanged.

The proposed procedure is conceived in such way that there is no concern about placing the sensors in an exact position. Moreover, considering that significant differences may be presented between any pair of IMU sensors, this setup allows the analysis of two different approaches when estimating the joint angles: (a) using the same sensor (IMU 3 or IMU 4) in different postures; and (b) using different sensors in the same posture. 

The orientation of each semi-sphere frame at any instant of time can be determined as qS1G, qS2IMU3G and qS2IMU4G. The joint J (Figure 2b) can be represented in a total of four different ways, as shown in Table 5. A simulation run in MATLAB under ideal conditions (IMU misalignment error, bias and noise equal to zero) is also presented to demonstrate that, theoretically, different orientations of IMUs do not affect the angle measures using the proposed method (assuming that IMUs 3 and 4 are ideally equal devices). In simulation, the initial orientation of the sensors was set to the initial values obtained during experimental validation. A demonstration can be seen on an online video (Supplementary Material)

### 3.3. Data Reduction and Statistical Analysis

A 20 min warm-up of the IMU sensors was carried out before the experiments, in an attempt to stabilize the gyroscope measurements [16]. After each rotation, the semi-sphere S2 was kept stationary approximately for 15 s. Only the last 10 s of collected data, for each orientation, were used. Quaternion for each rotation is resulted from averaging quaternion data over the 10 s intervals. Once data were reduced for each sensor and orientation, the IMU-to-body alignment method was applied to estimate the joint angles. Data were collected on two occasions, one day apart, and a total of twenty trials were acquired for each session. From trial to trial, IMUs 3 and 4 were exchanged of posture. These following approaches were statistically analyzed:

(1) In order to evaluate repeatability, understood as the consistency of measures of the IMU system under stated conditions on two days apart, a test-retest (intra-rater) study was performed. The angles, β, γ and δ were calculated for each representation of the joint and Intra-Class Correlation (ICC) was calculated. ICC (ICC(2,1),absolute agreement) was calculated using the software IBM SPSS Statistics 20 (α=0.05).
(2) In order to evaluate validity, root mean square error (RMSE) and concordance correlation coefficient (CCC, 95% IC) [28] between first-day measured joint angles (using IMU system) and reference values (using the gold-standard universal goniometer) were computed. Two scenarios are analyzed: (a) the differences of joint angles measures changing the postures (POS-1 and POS-2) of the sensors and (b) the differences of joint angles measures using different groups of sensors, i.e., IMU 3 relative to IMU 2 or IMU 4 relative to IMU 2, where IMUs 3 and 4 having the same posture in different occasions.

## 4. Application of the Calibration Procedure on Able-Bodied Subjects

### 4.1. Sensor Placement on Human Lower Limb

**Figure 3:**
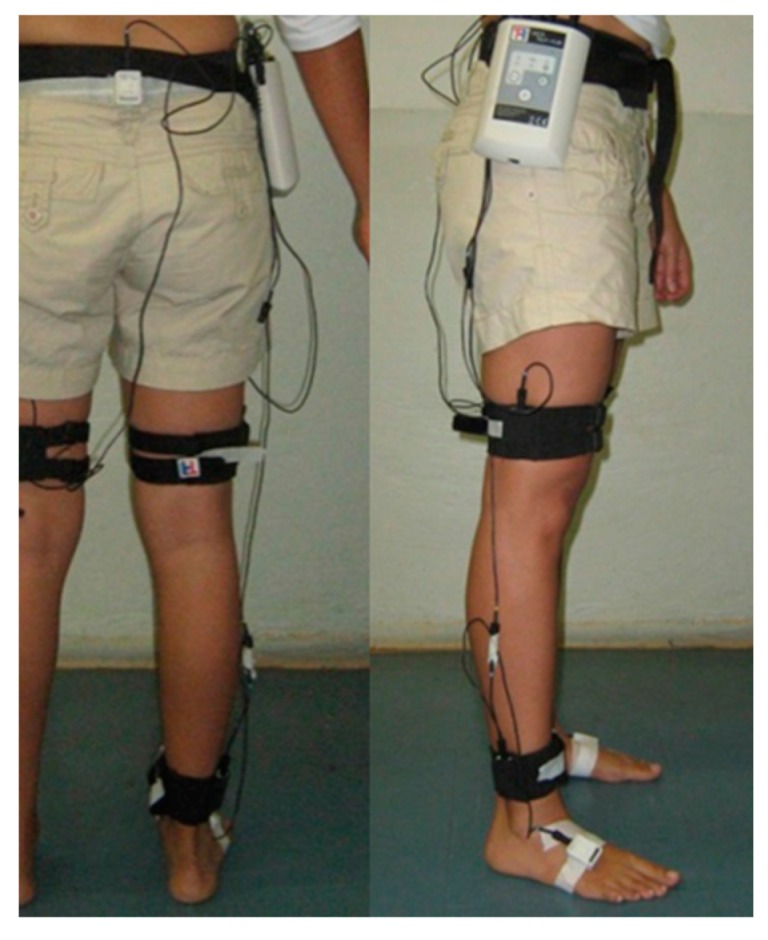
Sensor placement on the human lower limb.

Four sensors were positioned from the pelvis through the right lower limb (thigh, shank and foot segments, see Figure 3). The pelvis sensor was placed on the sacrum at the S2 spinous process in the middle point between two posterior superior iliac spines. The IMU describes a coordinate system defined as *x*-axis pointing cranially and *z*-axis pointing posteriorly. The thigh sensor was placed on the iliotibial tract approximately 5 cm above the patella. The shank sensor was positioned on the lower one-third of lateral shank 5 cm above of the lateral malleolus of the fibula.

The sensors on thigh and shank were positioned with *x*-axis pointing cranially and *z*-axis pointing laterally. The foot sensor was fixed with double sided tape on the dorsal region of the foot over the 3rd and 4th metatarsal bones, 3 cm above to the corresponding metatarsophalangeal joints, with *z*-axis pointing cranially and *x*-axis pointing posteriorly. These sensors were attached with double-sided tape on an acrylic plate, which was glued to elastic band with Velcro^®^. Similar positions have been suggested by different authors [10,14,29].

### 4.2. Discrete Parameters of the Joint Angles

Discrete angular kinematic parameters shown in Table 6 were estimated. Discrete parameters allow making a parametric analysis, which is demonstrated to be a reliable and practical method analyzing gait data, and it is a useful tool to assess data reliability [30]. These kinematic parameters were computed for each gait cycle. To determine them, there is a need to identify the two main phases of gait, stance and swing. This procedure of segmentation consists of determining the two events that indicate the start of each phase, which are heel strike (HS) and toe off (TO). Sabatini et al. [31] propose to determine HS and TO using the angular velocity sensed by a gyroscope on the foot. In our work, the orientation data of the foot were collected using quaternions. Each trial is divided in gait cycles to extract the kinematics parameters posteriorly. To determine the HS and TO, the angular velocity, as a function on quaternion, is computed as shown in Equation (9):

(9) Ωt=2qBF−FT*GS⊗q˙BF−FTGS,

where q˙BF−FTGS is the vector of quaternion rates (or the time derivate of the unit quaternion) of the foot, and $\Omega_{t}={\left(0,\omega_{x},\omega_{y},\omega_{z}\right)}^{T}$ is the quaternion representation of the angular velocity $\omega_{t}$. Using the component of the angular velocity on the sagittal plane ($\omega_{y}$, for IMU placed on the foot), the HS and TO events are determined using a minimum detection algorithm. In addition, with these two estimated events, the gait cycle is divided in the two main phases. Thus, it is possible to estimate the mentioned discrete kinematic parameters using maximum and minimum detection algorithm.

### 4.3. Experimental Protocol for Gait Analysis

Five volunteers without gait disabilities (two male and three female, 25 ± 4 years old) were enrolled in the validation procedure of this study. The IMU sensors were placed on pelvis and on right lower limb (thigh, shank and foot segments) by a trained physiotherapist as previously described in Section 4.1. The sensor placed on the pelvis was aligned with the walking direction. The subjects were asked to keep a straight upright posture during 5 s, and then walk 10 m in a straight line. Each subject performed three trials and the five middle gait cycles were extracted for analysis. This methodology was applied to ensure that only complete gait cycles were selected, excluding motion at the beginning and at the end of the walking process. Therefore, fifteen gait cycles were acquired for each subject. This research was approved by the Ethical Committee of UFES (Research Project 214/10).

## 5. Results and Discussion

This section presents the results of three approaches applying the proposed method: (1) A simulation that evidences the method performance regardless of drift errors and other perturbations associated with the IMU sensors (considering the limitations of the systems and applications that involve IMU sensors [12,13,14,15,16,22,32]); (2) a practical validation using an experimental simplified rigid-body joint and four IMU sensors; and (3) an application in human gait analysis. 

### 5.1. Simulation of the Proposed Method Applied to a Simplified Rigid-Body Joint

**Figure 4:**
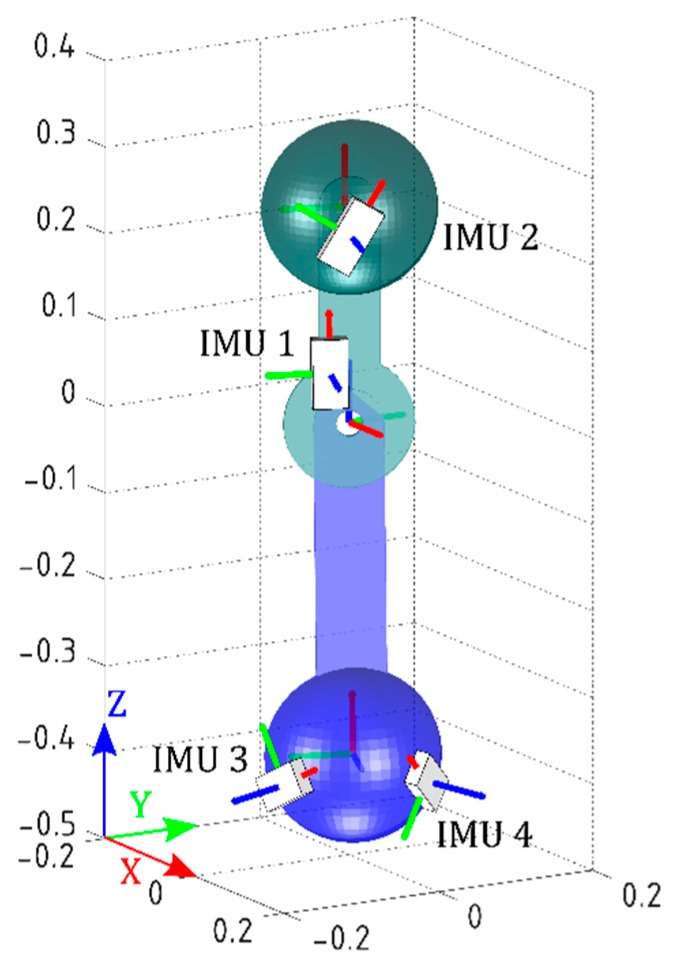
Simulation of the simplified joint. Scale models of the rigid-body joint and IMUs in MATLAB.

The IMUs’ initial orientations were set to the initial values obtained during practical validation, in order to run the simulation as close as possible to the real experiment. The models of the joint and the IMUs are shown in Figure 4. Movements from 0° to ±80° with steps of ±20° (called Postures 1 to 9) about *z*-axis of J were performed. Note that the simplified joint is analogous to a two-dimensional knee joint with one degree of freedom.

**Figure 5:**
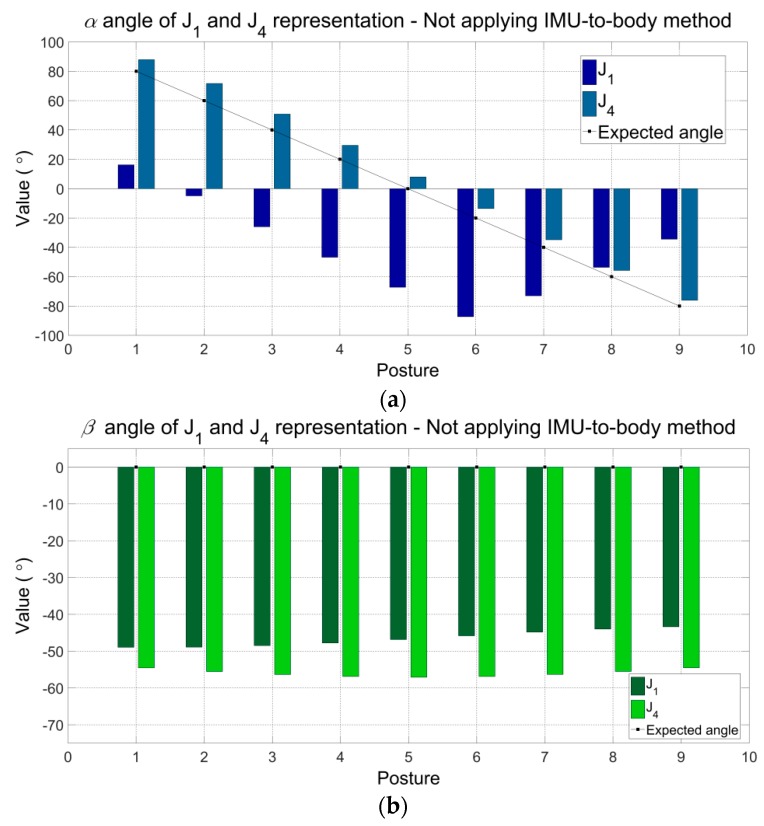

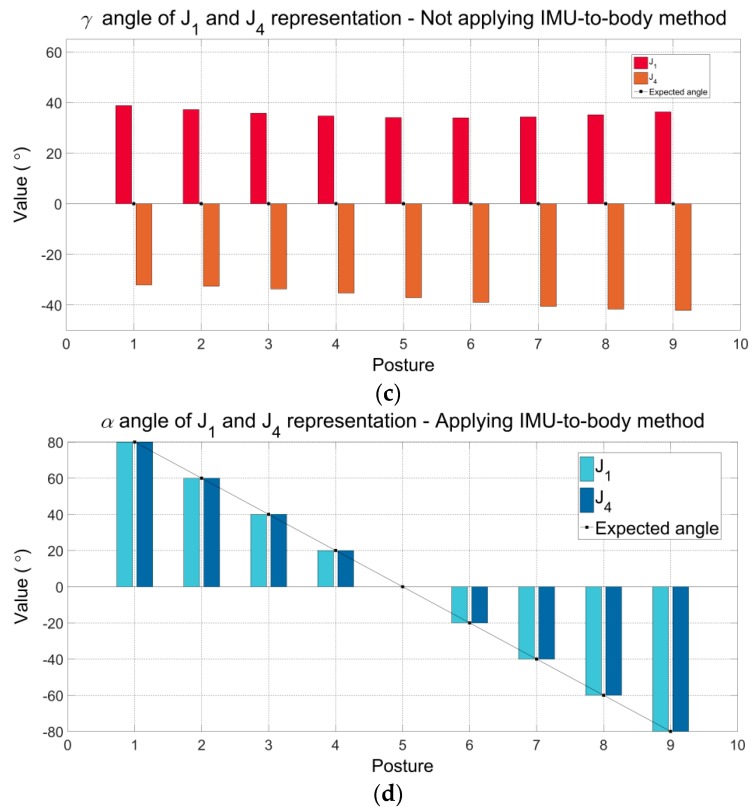
Comparison between the joint angles without applying the proposed procedure (**a**–**c**) and applying the procedure (**d**). Angular components *α*, *β* and *γ* are significant in the first case (**a**–**c**), which are different of the expected values. In the last case, only *α* is significant and equal to the expected values. *β* and *γ* are both equal to zero throughout the entire simulation, as expected. J_1_ and J_4_ are two representations of the simulated joint J represented by the goniometer.

Figure 5a–c show the angular components (α, β and γ) of the representations J_1_ and J_4_ (refer to Table 5) without applying the proposed method. Other representations of joint J present the same results. Because the proposed method was not yet applied, the angular components α, β and γ presented differences with the expected values. The maximum errors can be observed for J_1_: α (Posture 5) −67.26°, β (Posture 1) −48.96°, γ (Posture 1) 38.77°, and for J_4_: α (Posture 2) −11.69°, β (Posture 5) −57.09°, γ (Posture 9) −42.15.

After applying the proposed method, only α is significant under ideal conditions understanding that the rotations were applied exclusively around *z*-axis. Then, angular components β and *γ* are equal to zero. The angles α obtained by applying the IMU-to-body method are shown in Figure 5d. Notice that, as the angles β and γ are equal to zero, they are not graphically presented. Also, please observe that the values of *α* for J_1_ and J_4_ are equal to the expected values imposed by the simulation. In summary, through this simulation, we aim to demonstrate that applying the proposed method the estimated angles are equal to the expected values and consistent with the rotations applied. In addition, we also show that the proposed method produces the correct and consistent values when the IMU sensors are placed in different positions on the body segments.

### 5.2. Practical Validation of the Proposed Method Applied to a Simplified Rigid-Body Joint

Table 7 shows the data from ICC coefficients and its respective confidence intervals (95% IC) to evaluate the consistency of repeated measures of the IMU system under stated condition on two different days. ICC values were greater than 0.90 for all angular components and the different representations of the joint J. Movements associated with angles α, which correspond to flexion-extension angles on sagittal plane, produced the highest ICC values of the joint (ICC = 1.00). Observe that the angular component γ presented the lowest ICC values and the confidence intervals were wider (e.g., 0.60–0.97). We believe that such values are caused by limitations of the current IMU technology and fusion algorithms. The movements associated with *γ* angles correspond to external-internal rotation angles, which are performed on transversal plane, perpendicular to the gravity vector. In accordance with the literature, these movements around to the gravity vector present heading drift, which cannot be corrected using the accelerometer data. Therefore, this drift error may be associated with the performance of the magnetometer, gyroscope, and data fusion algorithm. Also, it has been mentioned that the heading drift is mainly due to the accuracy of the IMU sensors and, on a lesser extent, to the complexity of the task [33]. 

Table 8 and Table 9 report the Root Mean Square Error (RMSE) and Concordance Correlation Coefficient (CCC) obtained between first-day measured joint angles (using IMU system) and reference values (using the gold-standard universal goniometer) to evaluate validity, respectively.

The agreement between measures from IMU system and the universal goniometer applying the calibration procedure was excellent (CCC ≥ 0.98) for the angular component *α*. Note that for this angular component the maximum RMSE was 1.70° for the J_4_ representation on posture 2 (60°). Also, observe that the maximum RMSE (15.61°) is in correspondence with the angles γ. Again, these error drifts may be associated with the quality of the IMU data. In a previous validation study [34], the IMU sensors used here presented errors approximately up to 7° across 12 explored orientations, following the self-IMU consistency (SC) test. Errors were found up to 15°, following the Inter-IMU consistency (IC) test. These mentioned tests, with similar results, were proposed by Picerno et al. [16].

Note that the representations of J associated with IMU 3 (J_1_ and J_2_) presented lowest RMSE and highest CCC values broadly. It is possible to observe that for the angular component α, the measurements are not significantly different when using IMU 3 or IMU 4. However, for the angular components β and γ, the measurements using IMU 3 are lower than those using IMU 4. Additionally, using IMU 3 (the best case), RMSE values of β and γ apparently have similar magnitudes. Nevertheless, note that the magnitudes are not correlated with the same sense of rotation, it means that, for J_1_ representation (IMU 3: POS-1), errors are higher from 0 to −80°. On the other hand, for J_2_ representation (IMU 3: POS-2), errors are higher from 0 to 80°. Contrary to that demonstrated in simulation, the RMSE data suggest that the position of real IMU sensors is an important factor to consider in analyzes that involve the secondary planes of motion (coronal and transverse planes).

Besides, it is worth noting that the RMSE and CCC values mostly decrease as the angle increases. This can be observed for the angular components β and γ of the J_2_ representation. The angular component γ presented the lowest CCC values (0.02 ≤ CCC ≤ 0.05), however, note that for punctual cases, the CCC values were presented into acceptable to excellent interval. For example, for J_1_ representation between 80° to −20° (as highlighted in green color), the CCC values were from 0.48 to 0.99, corresponding with RMSE values smaller than 2.5°. This behavior may indicate that pairs of IMU sensors can be used on specific joints, according to their range of motion in gait analysis and, even in other applications that define limits of motion within the range of acceptable performance of the sensors. According to the results obtained using the simplified joint, we present in the next section the hip, knee and ankle joint angles in the sagittal plane through motion analysis using the proposed method. 

### 5.3. Experimental Validation for Gait Analysis

**Figure 6:**
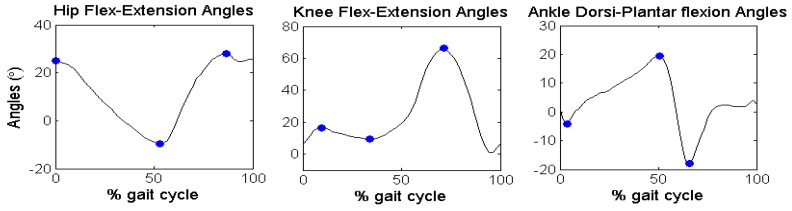
Discrete angular parameters on joint angles of Subject 2.

**Figure 7:**
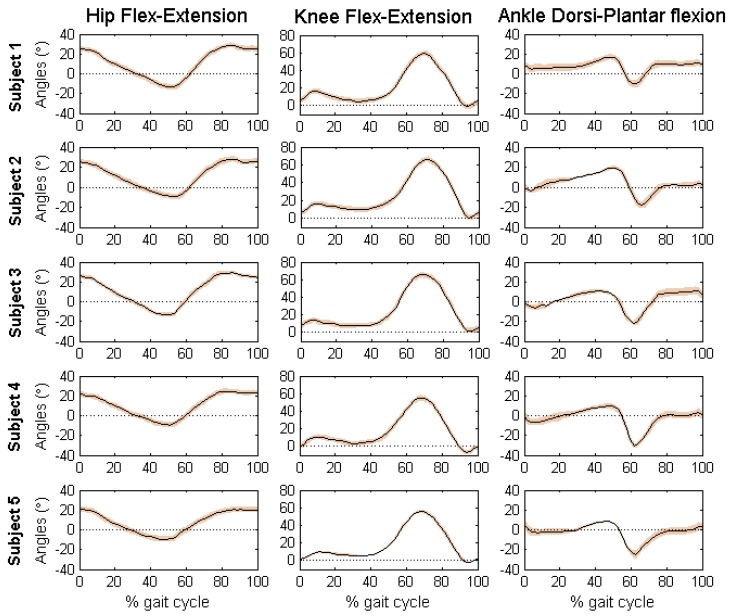
Joint angular kinematics in stride percentage (from HS to HS) of five able-body subjects. Fifteen gait cycles were summarized by black curve (MEAN) and orange stripe (±SD).

Figure 6 reports the discrete angular parameters (see Table 6) proposed for gait analysis of Subject 2, as an example, over one cycle of gait, to show graphically the kinematic parameters selected in the angular series. Figure 7 reports the mean and standard deviation of the joint angles in sagittal plane of the five volunteers. Table 10 shows the discrete angular parameters calculated using the mean of fifteen gait cycles for the five volunteers.

Mean and standard deviation of the joint angles of the five volunteers are within the normal range during a gait cycle for free walking. Interestingly, the results obtained with the developed algorithm presented low standard deviations, which means that estimated measures were consistent across trials. The maximum values of standard deviation were presented for the ankle joint angles of the five volunteers (Maximum SD = 3.99, AFE3, Subject 5). According to the results of each subject, it is possible to identify characteristics of each individual. By comparing the results obtained using the proposed method with the literature [12,15,29,30], it is clear that the angular patterns are coherent and within the intervals established by mean and standard deviations. It is important to highlight that these experiments were performed with the intention of proving a practical application of the proposed method. 

Notice that technical-anatomical frames, used to calculate the joint angles, are an estimate and may present a misalignment with the anatomical frame defined using bony landmarks. This means that joint angle curves may present an offset from values estimated using stereophotogrammetry, preserving the same angular patterns and range of motion.

## 6. Conclusions

In this work we have presented a novel calibration method to place and align inertial sensors with human body segments, with the goal of measuring joint angles. The advantages of the proposed method, in comparison with other methods described in the literature, include the fast and easy sensor placement, with no need of special movements performed by the user nor any additional tools, which may decrease setup time. The characteristics of this new method may make it more attractive for daily clinical routine. 

The results from the computational simulation demonstrate that, when applying the proposed method, the estimated angles are equal to the expected values and consistent with the joint’s rotations. Also, two real experiments have been carried out to evaluate the simulated procedure. Results indicate that the method is suitable to measure tridimensional angles of the hip, knee and ankle of the humans’ joints during free walking. However, some limitations mainly associated with the accuracy of the sensors used in the real experiments for practical validation gave rise to some estimation errors, mainly in movements around the gravity vector.

In conclusion, the proposed method is an interesting option to solve the alignment problem of human gait analysis based on inertial sensors. The discussed method is especially attractive for its simplicity and easy donning and doffing of the sensors. In applications such as gait rehabilitation, that requires motion analysis of impaired persons, the method can be of great help for its simplicity and accurate results.

## Figures and Tables

**Table 1:** Definition of technical-anatomical quaternions obtained during calibration posture (straight upright posture).

Segment	Initial Quaternion Definition
Pelvis (PV)	qBF−PVOGF
Thigh (TH)	qBF−THOGF=qBF−PVOGF⊗qROT(90°,i) 1
Shank (SH)	qBF−SHOGF=qBF−THOGF
Foot (FT)	qBF−FTOGF=qBF−SHOGF⊗qROT(180°,n2) 2

^1^ $i={\left[1\;0\;0\right]}^{T}$, ^2^ $n_{2}={\left[\sqrt{2}/2\;0\;\sqrt{2}/2\right]}^{T}$. Let $q_{ROT}\left(\theta ,n\right)$ be the quaternion of rotation calculated using Equation (5) for θ=90° or 180° and $n=i\;\mathrm{or}\;n_{2}$. BF refers to body-frame, GF to global frame.

**Table 2:** Body fixed, floating and references axes of each joint.

Joint	Joint Coordinate System	Body Fixed and Floating Axes	References Axes
HIP ^1^	Pelvis axis (flexion-extension)	$e_{1}=-y_{PV}$	$e_{1}^{r}=-z_{PV}$
	Femoral axis (internal-external rotation)	$e_{3}=x_{TH}$	$e_{3}^{r}=-y_{TH}$
	Floating axis (abduction-adduction)	$e_{2}=\frac{x_{TH}\times \left(-y_{PV}\right)}{\left|x_{TH}\times \left(-y_{PV}\right)\right|}$	
KNEE ^2^	Femoral axis (flexion-extension)	$e_{1}=z_{TH}$	$e_{1}^{r}=-y_{TH}$
	Tibial axis (internal-external rotation)	$e_{3}=x_{SH}$	$e_{3}^{r}=-y_{SH}$
	Floating axis (abduction-adduction)	$e_{2}=\frac{x_{SH}\times z_{TH}}{\left|x_{SH}\times z_{TH}\right|}$	
ANKLE ^1^	Tibial axis (dorsiflexion-plantar-flexion)	$e_{1}=z_{SH}$	$e_{1}^{r}=-y_{SH}$
	Calcaneal (internal-external rotation)	$e_{3}=z_{FT}$	$e_{3}^{r}=-x_{FT}$
	Floating axis (inversion-eversion)	$e_{2}=\frac{z_{FT}\times z_{SH}}{\left|z_{FT}\times z_{SH}\right|}$	

^1^ JCS proposed by Wu et al. [26] and ^2^ JCS proposed by Grood and Suntay [25]. PV pelvis, TH thigh, SH shank, FT foot.

**Table 3:** Rotations of the hip, knee and ankle joint of the right leg.

Joint	Flexion-Extension	Abduction-Adduction	Internal-External Rot
HIP	$\alpha =\mathrm{asin}\left(e_{2-H}\cdot x_{PV}\right)$	$\beta =\mathrm{acos}\left(-y_{PV}\cdot x_{TH}\right)-\frac{\pi }{2}$	$\gamma =\mathrm{asin}\left(e_{2-H}\cdot z_{TH}\right)$
KNEE	$\alpha =-\mathrm{asin}\left(e_{2-K}\cdot x_{TH}\right)$	$\beta =\mathrm{acos}\left(z_{TH}\cdot x_{SH}\right)-\frac{\pi }{2}$	$\gamma =\mathrm{asin}\left(e_{2-K}\cdot z_{SH}\right)$
ANKLE ^1^	$\alpha =\mathrm{asin}\left(e_{2-A}\cdot x_{SH}\right)$	$\beta =a\cos\left(z_{SH}\cdot z_{FT}\right)-\frac{\pi }{2}$	

^1^ Ankle rotations are dorsiflexion-plantar flexion and inversion-eversion. α, β and γ are the joint angles on sagittal, frontal and transverse planes, respectively. PV pelvis, TH thigh, SH shank, FT foot.

**Table 4:** Joint rotations as functions of quaternions.

Joint	Angles
HIP	α=asin(e2−H·M(qBF−PVGF)i)
	β=acos(−M(qBF−PVGF)j·M(qBF−THGF)i)−π2
	γ=asin(e2−H·M(qBF−THGF)k)
KNEE	α=−asin(e2−K·M(qBF−THGF)i)
	β=acos(M(qBF−THGF)k·M(qBF−SHGF)i)
	γ=asin(e2−K·M(qBF−SHGF)k)
ANKLE	α=asin(e2−A·M(qBF−SHGF)i)
	β=acos(M(qBF−SHGF)k·M(qBF−FTGF)k)

α, β and γ are the joint angles on sagittal, frontal and transverse planes, respectively. $e_{2-H}$, $e_{2-K}$ and $e_{2-A}$ are the floating axes of the hip, knee and ankle, respectively. M(qBF−XGF) is the equivalent direction-cosine matrix of the qBF−XGF quaternion of X body-segment. Body segments: PV pelvis, TH thigh, SH shank, FT foot.

**Table 5:** Representations of the J joint formed by S1 and S2 segments.

Joint	S1	S2	Posture
$J_{1}$	IMU 2	IMU 3	POS-1
$J_{2}$		IMU 3	POS-2
$J_{3}$		IMU 4	POS-1
$J_{4}$		IMU 4	POS-2

**Table 6:** Joint angles parameters for gait analysis.

Hip		Knee		Ankle	
Name	Variable	Name	Variable	Name	Variable
HFE1	Maximum hip flexion angle stance	KFE1	Maximum knee flexion angle stance	AFE1	Maximum ankle plantarflexion angle stance
HFE2	Maximum hip extension angle stance	KFE2	Maximum knee extension angle stance	AFE2	Maximum ankle dorsiflexion angle stance
HFE3	Maximum hip flexion angle swing	KFE3	Maximum knee flexion angle swing	AFE3	Maximum ankle plantarflexion angle swing

**Table 7:** Test-Retest study on two days apart: Consistency of measures of the IMU system.

Joint	Single Rater ICC Value					
	α		β		γ	
	Value	95% IC	Value	95% IC	Value	95% IC
$J_{1}$	1.00	1.00–1.00	0.99	0.98–0.99	0.95	0.83–0.99
$J_{2}$	1.00	1.00–1.00	0.99	0.99–0.99	0.96	0.88–0.95
$J_{3}$	1.00	1.00–1.00	0.98	0.96–0.99	0.90	0.60–0.97
$J_{4}$	1.00	1.00–1.00	0.99	0.99–0.99	0.99	0.98–0.99

**Table 8:** RMSE between the measurements from IMU system and the reference universal goniometer. Maximum RMSE values of each angular component are highlighted on orange color, and the acceptable values for angular components β and γ are highlighted on green color.

Joint	Angle	RMSE (°)									Max RMSE (°)
		80	60	40	20	0	−20	−40	−60	−80	
J_1_	α	0.67	0.64	0.49	0.30	0.07	0.48	0.74	0.90	0.93	0.93
	β	4.51	4.12	2.72	0.83	0.77	1.77	2.10	0.67	2.36	4.51
	γ	0.25	0.79	1.58	1.39	0.04	2.22	5.14	8.02	9.73	9.73
J_2_	α	0.60	0.47	0.16	0.22	0.04	0.49	1.03	1.31	1.21	1.31
	β	1.96	0.09	0.96	0.84	0.04	0.57	1.00	1.50	2.38	2.38
	γ	8.36	6.21	3.81	1.60	0.01	1.02	1.78	2.81	4.44	8.36
J_3_	α	1.41	1.13	0.68	0.43	0.02	0.24	0.38	0.39	0.23	1.41
	β	3.11	0.16	1.08	0.20	1.77	4.21	7.68	8.78	6.75	8.78
	γ	8.11	7.44	4.62	1.70	0.01	0.17	2.23	6.26	10.07	10.07
J_4_	α	1.42	1.70	0.94	0.55	0.08	0.27	0.63	0.90	0.60	1.70
	β	0.36	3.83	5.00	3.34	0.12	3.63	5.82	6.12	5.00	6.12
	γ	15.61	13.04	7.53	2.69	0.04	0.09	1.74	3.96	5.50	15.61

**Table 9:** CCC between the measurements from IMU system and the reference universal goniometer. Minimum CCC values of each angular component are highlighted on orange color, and the acceptable values for angular components β and γ are highlighted on green color.

Joint	Angle	CCC ($\rho_{c}$)									Min. CCC
		80	60	40	20	0	−20	−40	−60	−80	
J_1_	α	0.99									0.99
	β	0.25 *	0.28 *	0.49 ^†^	0.94	0.90	0.67	0.67	0.99	0.49 ^†^	0.25 *
	γ	0.87	0.72	0.58	0.69	0.99	0.48 ^†^	0.16 *	0.07 *	0.05 *	0.05 *
J_2_	α	0.99									0.99
	β	0.53	0.88	0.66	0.77	0.99	0.87	0.67	0.51 ^†^	0.40 ^†^	0.30 *
	γ	0.05 *	0.09 *	0.20 *	0.60	0.99	0.81	0.61	0.40 ^†^	0.24 *	0.05 *
J_3_	α	0.99									0.99
	β	0.31 *	0.99	0.97	0.99	0.97	0.28 *	0.12 *	0.10 *	0.16 *	0.10 *
	γ	0.06 *	0.08 *	0.24 *	0.99	0.99	0.99	0.48 ^†^	0.17 *	0.04 *	0.04 *
J_4_	α	0.98									0.99
	β	0.97	0.30 *	0.19 *	0.35 *	0.99	0.32 *	0.16 *	0.14 *	0.18 *	0.14 *
	γ	0.02 *	0.04 *	0.11 *	0.49 ^†^	0.99	0.99	0.68	0.29 *	0.17 *	0.02 *

* Less than 0.40: agreement between measure poor. ^†^ Between 0.40 and 0.59: agreement fair.

**Table 10:** Mean (SD) of the discrete parameters reported for five volunteers.

Parameter	MEAN (SD) (°)				
	Subject 1	Subject 2	Subject 3	Subject 4	Subject 5
HFE1	25.44 (2.62)	25.14 (2.78)	26.19 (2.18)	23.18 (2.54)	20.75 (2.73)
HFE2	−13.52 (3.97)	−9.62 (2.81)	−13.39 (2.00)	−9.39 (2.67)	−9.66 (2.86)
HFE3	28.87 (2.51)	27.96 (2.57)	29.38 (1.83)	24.63 (2.44)	20.28 (2.73)
KFE1	16.24 (3.10)	16.29 (2.75)	14.08 (3.12)	10.79 (2.65)	9.99 (0.63)
KFE2	4.63 (2.98)	9.62 (2.92)	7.58 (2.98)	2.98 (3.20)	5.23 (0.23)
KFE3	59.35 (1.70)	66.24 (2.82)	65.59 (2.88)	55.01 (2.80)	55.93 (1.55)
AFE1	−1.42 (3.93)	−4.16 (1.56)	−6.08 (2.51)	−6.76 (3.42)	−2.93 (3.98)
AFE2	16.95 (2.51)	19.43 (1.21)	10.55 (3.74)	9.53 (2.63)	8.48 (1.64)
AFE3	−10.32 (3.52)	−17.80 (3.46)	−21.52 (2.02)	−30.51 (2.49)	−25.15 (3.99)

## Supplementary Materials

A demonstration of the simulation can be seen on an online video: https://www.dropbox.com/s/gu8w5wbz7ofcztp/SimulationIMU.mp4?dl=0.
